# Sources of Variation in Cuticular Hydrocarbons in the Ant *Formica exsecta*

**DOI:** 10.1007/s10886-013-0366-0

**Published:** 2013-11-24

**Authors:** Stephen J. Martin, Emma Vitikainen, Sue Shemilt, Falko P. Drijfhout, Liselotte Sundström

**Affiliations:** 1 School of Environmental and Life Sciences, University of Salford, Greater Manchester, M5 4WT UK; 2 Centre of Excellence in Biological Interactions, Department of Biosciences, University of Helsinki, P.O. Box 65, 00014 Helsinki, Finland; 3 Present Address: Centre for Ecology and Conservation, University of Exeter, Penryn Campus, Exeter, TR10 9EZ UK; 4 Chemical Ecology Group, School of Physical and Geographical Sciences, Lennard-Jones Laboratory, Keele University, Keele, ST5 5BG UK

**Keywords:** Alkanes, Alkenes, Cuticular hydrocarbons, Recognition, Phenotype, Chemotype, Variation

## Abstract

Phenotypic variation arises from interactions between genotype and environment, although how variation is produced and then maintained remains unclear. The discovery of the nest-mate recognition system in *Formica exsecta* ants has allowed phenotypic variation in chemical profiles to be quantified across a natural population of 83 colonies. We investigated if this variation was correlated or not with intrinsic (genetic relatedness), extrinsic (location, light, temperature), or social (queen number) factors. (*Z*)-9-Alkenes and *n*-alkanes showed different patterns of variance: island (location) explained only 0.2 % of the variation in (*Z*)-9-alkenes, but 21–29 % in *n*-alkanes, whereas colony of origin explained 96 % and 45–49 % of the variation in (*Z*)-9-alkenes and *n*-alkanes, respectively. By contrast, within-colony variance of (*Z*)-9-alkenes was 4 %, and 23–34 % in *n*-alkanes, supporting the function of the former as recognition cues. (*Z*)-9-Alkene and *n*-alkane profiles were correlated with the genetic distance between colonies. Only *n*-alkane profiles diverged with increasing spatial distance. Sampling year explained a small (5 %), but significant, amount of the variation in the (Z)-9-alkenes, but there was no consistent directional trend. Polygynous colonies and populous monogynous colonies were dominated by a rich C_23:1_ profile. We found no associations between worker size, mound exposure, or humidity, although effect sizes for the latter two factors were considerable. The results support the conjecture that genetic factors are the most likely source of between-colony variation in cuticular hydrocarbons.

**Electronic supplementary material:**

The online version of this article (doi:10.1007/s10886-013-0366-0) contains supplementary material, which is available to authorized users.

## Introduction

Disentangling the various genetic and environmental factors that contribute to phenotype variability in out-bred natural populations is a major challenge (Peaston and Whitelaw 2006). Genotype-environment interactions cause phenotypic variation in both vertebrates (Lindström 1999) and invertebrates (e.g., Bonasio et al. 2011; Vogt et al. 2007; Xiang et al. 2010). Most animals rely on recognition, be it chemical, visual, or acoustic, in order to identify friends from foes correctly. Insects predominantly use chemical signals to communicate for a wide range of behaviors (Blomquist and Bagnères 2010; Wyatt 2003). Yet, the degree to which chemical phenotype, or ‘chemotypic’, variation exists within any natural social-insect populations remains poorly understood, partly because chemical profiles contain a multitude of signals (Hefetz 2007) that are not readily differentiated by researchers. The difficulty in identifying the precise chemicals used by insects for these signals, such as recognition of conspecifics, has led to the use of multivariate statistics to help define their composition (e.g., Kather et al. 2011; Lahav et al. 2001). Without differentiation of the signals, it is impossible to establish the natural range of chemotypic variation that exists in a population, and how different genetic and environmental factors determine an individual’s chemotype.

It is well established that cuticular hydrocarbon (CHC) production in insects is under genetic control (Wicker-Thomas and Chertemps 2010) and that these chemicals are involved in recognition. A strong genotype-phenotype link in the production of CHC’s is assumed to exist in social insects, such as ants, bees, termites, and wasps, in order to ensure that altruistic acts are directed toward kin. Although several studies have found a predicted genotype-chemotype link in bees (e.g., Arnold et al. 1996; Greenberg 1979), other studies suggest that recognition is not based solely upon heritable characteristics (e.g., Adams 1991; Gamboa 2004). However, in these and other studies, the actual recognition systems used by the insects has not been chemically defined. This lack of definition of a chemical profile has been a major obstacle in defining the range of chemotypic variation and studying how various intrinsic and extrinsic factors affect this variation.

**Fig. 1:**
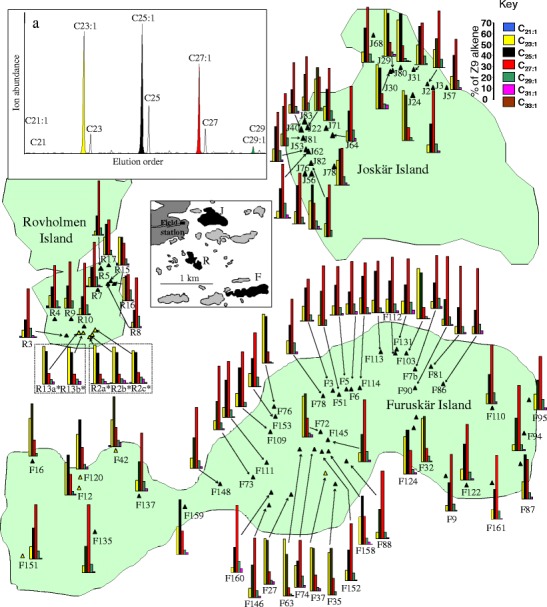
**a** Total ion chromatogram of a *Formica exsecta* worker showing the typical series of *n*-alkane and (*Z*)-9-alkenes. The locations and (*Z*)-9-alkene profiles of the 86 mounds (83 colonies) chemo-typed in 2008 are shown on the three study islands. Polygynous mounds are indicated by a *white* (printed) or *yellow* (online) *triangle* and mounds belonging to the same colony are grouped within a *dotted line*. The location of each island relative to the field station is given in the insert. The distance between Joskär and Rovholmar is 800 m, between Joskär and Furuskär, 1500 m, and between Furuskär and Rovholmar, 1000 m

The study species *Formica exsecta* Nylander is a territorial ant species, widely distributed across the Palaearctic region, where it inhabits meadows and forest clearings (Czechowski et al. 2002). This species occurs in two distinct social forms: colonies with a single egg-laying queen (monogynous) that are founded through temporary social parasitism of other *Serviformica* ant species, and colonies with multiple queens (polygynous) that presumably arise from monogynous colonies through adoption of daughter queens. The chemical profile of this species has been extensively studied across Europe (Martin et al. 2008a, b, 2009, 2011, 2012a, b, c, 2013; van Zweden et al. 2011), demonstrating a simple and consistent CHC profile, composed of homologous series dominated by three *n*-alkanes (C_23_, C_25_, and C_27_) and three (*Z*)-9-alkenes (C_23:1_, C_25:1_, and C_27:1_), with a fourth (C_29_ and C_29:1,_ respectively) of each series always present in lower quantities (Fig. 1a). Only the (*Z*)-9-alkenes have been shown to act as nest-mate recognition cues (Martin et al. 2008a, 2012b), with changes in *n*-alkanes corresponding with task differences in *F. exsecta* (Martin and Drijfhout 2009), other ants (Wagner et al. 1998), and honeybees (Kather et al. 2011).

After the elucidation, based on 10 colonies, of the recognition system of *F. exsecta* (Martin et al. 2008a, b), we decided to chemotype 83 colonies, in 2008, across three study islands, located near the Tvärminne zoological station in Hanko, Finland. The aims of this study were to quantify the amount of chemotypic variation in (*Z*)-9-alkene and *n*-alkane profiles across the 83 colonies, and to search for a series of intrinsic and extrinsic factors that might contribute to the observed pattern of profile variation. For intrinsic factors, we included 72 colonies, and investigated whether genetic and chemical distances were correlated. For extrinsic factors, we studied temperature and humidity, since these can affect the CHC profile of ants (Martin and Drijfhout 2009; Wagner et al. 1998). Chemotypic variation in some species of *Formica* ants also may be affected by social factors, such as parasite pressure (Martin et al. 2011), invasiveness (Errard et al. 2005), and queen number; e.g., in *F. exsecta* (Martin et al. 2009) and *F. truncorum* (Johnson and Sundstrom 2012), but not in *F. fusca* (Helanterä et al. 2011). Therefore, we also investigated factors, such as queen number, colony size, and age (the latter two are correlated with queen number). Finally, it is known that the CHC profile of ants fed artificial diets under laboratory conditions can change, due to acquisition of novel prey-specific compounds (i.e., via contamination; Liang and Silverman 2000), or through the relative proportions of carbohydrates and protein in the diet (Buczhowshi et al. 2005; Sorvari et al. 2008). *Formica spp.* are generalists, feeding on both honeydew (carbohydrate) and an array of invertebrates (Skinner 1980). Therefore, we tested whether diet is an important determinant of CHC variation in this species, by examining spatial and seasonal changes of the CHC profile, especially in the part of the CHC profile (*n*-alkanes) not associated with nest mate recognition.

## Methods and Materials

### Study Population

**Fig. 2:**
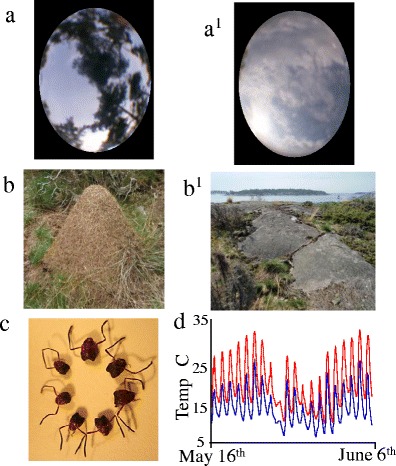
Photographs illustrating the range in: **a**–**a** ^**1**^ mound exposure, **b**–**b** ^**1**^ colony size and aspect, **c** head size, and (**d**) mound temperatures, found across the study population of *Formica exsecta*

The 83 colonies included in this study were located on three islands, Joskär (10-ha), Rovholmar (5-ha), and Furuskär (30-ha), all adjacent to the Tvärminne Zoological station, on the Hanko peninsula in Southern Finland (Fig. 1). The populations have been surveyed yearly for colony births and deaths, as well as for colony size (number of workers), since 1993 on Joskär (Chapuisat et al. 1997; Sundström et al. 1996, 2003), and 2000 on Furuskär (Haag-Liautard et al. 2009; Vitikainen and Sundström 2011; Vitikainen et al. 2011). The third island, Rovholmar, was colonized after 2000 and has been monitored since 2005 (Haag-Liautard et al. 2009; Vitikainen et al. 2011). Thus, the age, size, and genetic structure of each colony was known. All *F. exsecta* colonies were marked the first time they were found with a unique number, and their position recorded using GPS (Garmin eTrex). All colonies had been genotyped at ten DNA microsatellite loci (Haag-Liautard et al. 2009; Vitikainen et al. 2011), yielding a large database of all colony genotypes that is continually maintained and updated. In the study population, 90 % of colonies were monogynous, 83 % of which were monandrous; the remaining 10 % were polygynous. Thus, as for other populations (Rosengren et al. 1993; Seppä et al. 2004), one social form (monogynous) dominates. As queens mate for life and the number of mother queens in monogynous colonies remains stable throughout the entire life of a colony, measures of relatedness remain the same irrespective of when they were chemo-typed. This has been ascertained by re-genotyping colonies across years. Colony sizes during the summers of 2008 and 2010 were estimated following the procedures described in Haag-Liautard et al. (2009) and Vitikainen et al. (2011), including the sample-size correction suggested by Pollock et al. (1990). Spatial separation was measured as the geometric distance between all pairs of colonies, assuming sexuals can fly directly across open water when dispersing from one island to another. Within the study populations, there is noticeable variation between colonies in worker size (Fig. 2c), which may reflect colony age or more favorable rearing conditions. Therefore, we measured the head width of ten ants from each colony using a Leica binocular microscope fitted with a graticule. The mean head width for each colony was calculated to the nearest 0.1 mm and used as a covariate in the GLM analyses. As workers are produced in one cohort each year, and live around 1 year (Vitikainen 2010), the amount of within-colony variation in worker size within each season is low.

### Measurements of Environmental Factors

Although *F. exsecta* nest in open areas, there is extensive variation in the amount of cover from grass and trees surrounding each mound (Fig. 2a, b). We measured the amount of light reaching a mound (exposure) on sunny days with little or no cloud cover by using a 120° fish eye lens attached to a digital camera (Olympus FE-230), with the image looking directly up from the surface of the mound. The image was converted into black and white, and the proportion of white pixels calculated. The resulting index was taken to reflect mound exposure, and was used as a covariate in the GLM analyses. All image processing and analyses were conducted using ImageJ (http://rsbweb.nih.gov/ij/).

To quantify variation in mound temperature, we placed temperature loggers (Thermochron iButtons), from August 6th to September 2nd 2009, into the mounds of ten colonies that covered the entire range of (*Z*)-9-alkene profiles found in the populations. Each logger was placed 5 cm below the mound surface, and logged temperature every hour. This was also carried out from 16th May to 1st September 2010 and again from 20th April to 15th June 2011. These data were used to calculate the average mound temperature over each study period and over the three study years for each colony. During both 2009 and 2010, nine loggers also recorded the relative humidity every hour in nest mounds. Again, the average relative humidity was calculated for each mound, separately for the 2 years, and overall.

### Chemotyping Procedures

During September 2008, ten workers were collected from each of the 83 colonies. Thirty one of these colonies were re-sampled in August 2010 to investigate the effect of time on profile stability over the study period. Ants were frozen and stored at −20 °C until chemically analyzed. CHCs were extracted from each ant in 30 μl of HPLC-grade hexane and analyzed on an Agilent 7890 gas chromatograph (GC) connected to an Agilent 5975 MSD (quadropole) mass spectrometer (MS; −70 eV, electron impact ionization). The GC was equipped with a ZB-5HT column (length, 30 m; ID, 0.32 mm; film thickness, 0.25 μm; Phenomenex, Torrance, CA, USA), and the oven temperature programmed from 50 to 110 °C at 40 °C.min^−1^ and then to 360 °C at 20 °C.min^−1^. Samples were injected in splitless mode, with helium as carrier gas, at a constant flow of 1.0 ml.min^−1^. Hydrocarbons were characterized using diagnostic ions and Kovats indices. We had already established that no high molecular weight hydrocarbons were present on *F. exsecta* (unpublished data) using high temperature GC/MS methods (Sutton et al. 2013).

The peak area of each hydrocarbon was integrated manually from the total ion chromatogram, and then the proportion of each (*Z*)-9-alkene or *n*-alkane calculated using the total amount of (*Z*)-9-alkenes or *n*-alkanes, respectively. Five (*Z*)-9-alkenes (C_23:1_–C_31:1_) and five *n*-alkanes (C_23_–C_31_) were identified, but in both cases the C_31_ compounds were present only in trace amounts in <50 % of individuals. These C_31_ compounds were omitted from all analyses. In addition to the three dominant compounds each of (*Z*)-9-alkanes and *n*-alkenes included in earlier studies, a fourth one (either C_29:1_ or C_29_) of each was present in low amounts in all samples, and was included in the analyses. Three percent of samples had low ion counts (<1E+7) and were discarded, as low concentrations can distort the profile. The (*Z*)-9-alkene or *n*-alkane colony profile was determined from the ten workers collected in 2008, and from five workers collected in 2010, for each colony.

### Statistical Analyses

Peak areas were log normalized using the Aitchison transformation (Aitchison 1986). We then used principal component analysis (PCA) to reduce the number of variables and to identify peaks that contributed greatest to the variation along each axis. We analyzed the apportionment of variance and tested for differences among islands and colonies by nested ANOVA, with colonies nested within islands. To estimate the association between CHC profiles and genetic and spatial distance, we constructed a distance matrix based on squared Mahalanobis distances between group centroids across colonies. These estimates were implemented in the Mantel tests described below. Finally, to assess (*Z*)-9-alkene and *n*-alkane profile stability in the subset of colonies that were sampled in both study years (*N* = 33), we examined the variance components (REML) among colonies and between years within colonies. We followed this up with a linear regression on mean colony factor scores in the two study years and examined both the association between profiles between years, and the intercept for trends in the direction of change. These analyses were carried out in Statistica 10. Throughout, all analyses were carried out separately for (*Z*)-9-alkenes and *n*-alkanes.

Based on the existing genotype data in our database, we calculated the corresponding genetic distance for each pair of colonies, as the pair-wise fixation index (F_ST_), using the program Fstat 2.9.3.2 (Goudet 1995, 2001). The relationships between colony-specific profiles [(*Z*)-9-alkenes and *n*-alkanes], and spatial and genetic distance between every pair of colonies were tested using a Mantel test (MantelTester v. 1.0, Bonnet and Van de Peer 2002). All significant results were followed up with Partial Mantel tests controlling for each of the other two factors.

We used general linear models (GLM) to test for environmental (mound exposure) and colony-specific (colony type, queen number, within-colony relatedness, colony size, and worker head size) effects on colony chemical profile, as represented by the mean of the factor scores of the (*Z*)-9-alkene or *n*-alkane profile. In addition, we used partial correlations to test for effects of temperature and humidity in a subset of colonies. Colony size, head width, and shading were standardized to improve normality of residuals, which was verified by examining residual plots as described in Zuur et al. (2007). The full model, including all first-order interactions, was evaluated, and then simplified by dropping non-significant terms (*P* > 0.15), sequentially, to achieve the minimal model with only significant terms (*P* < 0.05). Each dropped term was retested by adding it back into the final model, in order to verify that the (non-) significance of the term was not contingent on the order in which it was removed from the model. These statistical analyses were conducted in GenStat 12.2.0.3717, VSN International Ltd.

## Results

### Genetic Population Structure and Variation in Chemical Profile

The distribution and range of colony-specific profiles of the 83 colonies sampled in 2008 indicated a wide range of (*Z*)-9-alkene (C_23:1_–C_29:1_; Fig. 1) and to a lesser extent *n*-alkane (C_23_–C_29_) (data not shown) chemotypes, both across the entire population and on each island. The first factor (PCA) explained 80 % of the total variation in the four (*Z*)-9-alkenes, but only 59 % of the total variation of the four *n*-alkanes. The loadings for the first factor (PCA) for C_23:1_ and C_25:1_ were negative (−0.92 and −0.89, respectively), whereas those for C_27:1_ and C_29:1_ were positive (0.94 and 0.84, respectively). For the (*Z*)-9-alkenes, between-colony variation explained 96 % (ANOVA with colony nested within island: *F*_81,669_ = 191.1, *P* < 0.001), between-island variation explained 0.2 % (*F*_2,81_ = 0.07, *P* = 0.93), and within-colony variation (error variance) explained only 4 %, of the total variation along the first factor. Individual compounds all produced the same outcome (Supplemental Table 1). For the *n*-alkanes, the between-colony differences explained 45 % of the variation along the first axis (ANOVA with colony nested within island, *F*_82,713_ = 13.93, *P* < 0.001), between-island variation explained 21 % of the variation (*F*_2,82_ = 8.72, *P* < 0.001), and within-colony variance (error) explained 34 % of the variation; the last mentioned was considerably higher than that for the (*Z*)-9-alkenes. Again, individual compounds produced largely similar outcomes, except that C_27_ produced no among-island differences (Supplementary Table 1).

Chemical, spatial and genetic data were available for 72 colonies across the three islands. Both (*Z*)-9-alkenes and *n*-alkanes chemical distances increased with increasing genetic distance [partial Mantel tests, corrected for spatial distance, (*Z*)-9-alkenes: *r* = 0.163, *P* = 0.01; *n*-alkanes: *r* = 0.195, *P* = 0.003]. For (*Z*)-9-alkenes, spatial distance did not correlate with chemical distance (*r* = 0.019, *P* > 0.05), but for *n*-alkanes, the chemical distance increased with spatial distance (*r* = 0.143, *P* = 0.002). No correlations were found between spatial and genetic distance for either type of compound (*r* = −0.025 and 0.027, respectively, *P* > 0.05).

Analysis of the variance components for colonies sampled both in 2008 and 2010 revealed a year effect for both (*Z*)-9-alkenes and *n*-alkanes. Colony of origin explained 91 % of the total variation in the (*Z*)-9-alkene factor scores (REML colony: *z* = 3.81, *P* < 0.001), and year of sampling also explained a significant, although small, proportion (5 %) of the variation within each colony (REML: colony * year *z* = 3.59, *P* < 0.001; residual variance: 4 %, *z* = 13.9, *P* < 0.001). Individual compounds gave the same pattern (Supplemental Table 2). A linear regression of the colony mean factor score for 2010, on that for 2008, revealed a very close between-year relationship between colony-specific factor scores (*R* ^2^ = 0.88, *F*_1,30_ = 221.97, *P* < 0.001), but no significance for the intercept (*b* = −0.04, SE = 0.06, *t*_30_ = −0.63, *P* = 0.52).

For *n*-alkanes, the variance between sampling years was significant, explaining 41 % and 54 % of variance in the first two mean factor scores, respectively (REML year*colony: *z* = 3.55, *P* < 0.001 and *z* = 3.78, *P*, = 0.001). Colony of origin only explained 20 % and 19 % of the total variation (REML colony: *z* = 1.58, *P* = 0.11, and *z* = 1.35, *P* = 0.18; residual variances, including between-island variation: 39 % and 26 %, respectively, *z* = 14.44 *P* < 0.001 in both cases). All four compounds, individually, also gave a very similar pattern (Supplemental Table 2). In contrast to (*Z*)-9-alkenes, we found no association between colony-specific *n*-alkane factor scores between years (*R* ^2^ = 0.09, *F*_1,31_ = 2.99, *P* = 0.09, and *R* ^2^ = 0.06, *F*_1,31_ = 2.11, *P* = 0.15). As for the (*Z*)-9-alkenes, the intercept was not significant (*b* = 0.01, SE = 0.11, *t*_31_ = 0.12, *P* = 0.90, and *b* = 0.003, SE = 0.10, *t*_30_ = 0.04, *P* = 0.97, respectively). The same pattern applied for each compound individually (Supplemental Table 2).

### The Impact of Social and Environmental Factors on Chemical Variation

**Fig. 3:**
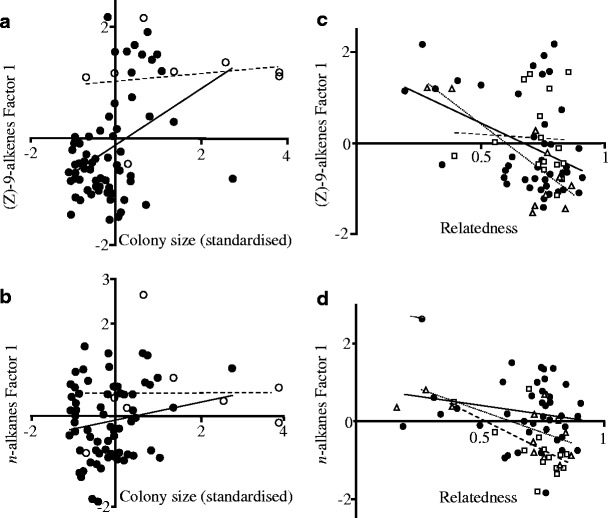
Effect of colony size on, **a** (*Z*)-9-alkene Factor 1 score, and **b**) *n*-alkanes Factor 1 score (monogyne colonies are represented by *solid circles* and *lines*, while polygyne colonies are represented by *open circles* and *dashed lines*). The effect of relatedness and island on, **c** (*Z*)-9-alkenes, and **d**) *n*-alkanes. Factor 1 scores are shown. *Solid circles* and *lines* indicate colonies from Furuskär, *open squares* and *dashed lines* indicate colonies from Joskär, and *open triangles* and *dotted line* indicate colonies from Rovholmama Island

Seven of the study mounds were polygynous and 79 monogynous. Social type (i.e., monogynous or polygynous) explained a borderline significant amount of variation in the (*Z*)-9-alkene profile (GLM on mean colony factor scores: *F*_1,71_ = 3.87, *P* = 0.05), with polygynous colonies showing a preponderance of shorter-chain compounds; i.e., they had a C_23:1_-rich profile. Given that colony size also had an effect on the chemical profile (*F*_1,71_ = 23.1, *P* < 0.001; Fig. 3a, b), and that the interaction between social type and colony size was significant (*F*_1,71_ = 4.35, *P* = 0.04), this effect was partly due to differences in colony size between the two social types. Thus, when combining the information from the factor scores for each compound and colony type/size, shorter-chain compounds dominated in polygynous colonies, as well as more populous monogynous colonies, whereas longer-chain compounds dominated in less populous monogynous colonies. In the case of *n*-alkanes, we entered island as a main effect in the model, as the earlier analysis indicated differences among islands for both factors. Factor 1 scores also decreased with increasing within-colony relatedness (*F*_1,68_ = 9.07, *P* = 0.004; Figs. 3c, d), indicating a preponderance of longer-chain compounds in colonies with high-relatedness (e.g., monogynous).

Worker head width varied among colonies from 1.107 to 1.508 mm (Fig. 2c), but within-colony variation was low (pooled SD = 0.075 mm). Regardless of social type, older and larger colonies had larger workers, yet workers in polygynous colonies were smaller than those in monogynous colonies (Supplemental Table 3), even though polygynous colonies were, on average, more populous [mean = 5451 ± 1423 (±SD) ants] than monogynous ones (1852 ± 164 ants; *t*_78_ = −5.327, *P* < 0.001). Mound exposure ranged from completely exposed (<1 % cover) to largely shaded (up to 62 % cover; Fig. 2a), with an average exposure of 24 ± 14 % (±SD; *N* = 95 mounds). This indicated that most mounds were relatively exposed, which is typical for this species. Larger colonies were more shaded (*R* ^2^ = 0.236, *P* = 0.043, *N* = 74), but there was no effect of shading on worker size (*F*_1, 68_ = 1.45, *P* = 0.23). We found no association among worker size, mound exposure, nor any of the other tested variables or their interactions, and the (*Z*)-9-alkene factor scores (Supplemental Table 4 and 6a). None of the other factors had an effect on *n*-alkane profile (Supplemental Table 5 and 6b).

The average temperature across the ten colonies in which temperature was measured ranged from 11.7 to 16.6 °C in spring (2011), 16.4–22.8 °C in summer (2010) and 18.1–21.7 °C in autumn (2009). The average temperature of colonies was highly correlated among study years (*r* = 0.89, *P* < 0.001, *N* = 10), which suggests that colonies had different microclimates. Like temperature (Fig. 2d), humidity had a daily cycle varying from 50 to 100 %; contrary to temperature, humidity was not correlated among study years (*r* = 0.34, *P* = 0.36, *N* = 9). Average temperature and humidity were not correlated (*r* = −0.58, *P* = 0.08, *N* = 10), and neither was correlated with colony exposure (temperature, *R* = −0.39, *P* = 0.27, *N* = 10; humidity, 0.37, *P* = 0.29, *N* = 10). Neither colony temperature nor humidity correlated with the (*Z*)-9-alkenes (Partial correlation with factor scores, correcting for humidity, *R* = 0.59 *P* = 0.06, *N* = 11, and correcting for temperature, *R* = 0.43, *P* = 0.18, *N* = 11; Supplemental Figs. S1 a,c), or with the *n*-alkanes (Partial correlation correcting for humidity, Factor 1, *r* = 0.48, *P* = 0.14, *N* = 11, and for temperature, *R* = 0.34, *P* = 0.31, *N* = 11; Supplemental Figs. S1b, d). Nonetheless, the effect sizes were considerable.

## Discussion

As expected, chemotypic variation among colonies was very high, both across the entire population and on each island. For (*Z*)-9-alkenes, between-colony variation accounted for 96 % of the total variation, and within-colony variation accounted for only 4 %. By contrast, for *n*-alkanes, between-colony differences explained 45 % of the variation and within-colony variance (error) 34 %. Moreover, for a given colony, the (*Z*)-9-alkene profile was consistent, whereas the *n*-alkane profile was more variable among years. This supports an earlier interpretation that the (*Z*)-9-alkene profile conveys information on colony identity (Martin et al. 2008a, b), whereas the *n*-alkane profile is less tightly linked to colony affiliation and, together with (*Z*)-9-alkenes, provides an anti-desiccation function.

On a larger spatial scale, across islands, we found no segregation of (*Z*)-9-alkenes and significant segregation for *n*-alkanes, yet we found correlations between genetic and chemical distance for both groups of compounds, such that chemical distance increased with genetic distance. This suggests a genetic influence on the CHC profile, as demonstrated earlier in a smaller data set (van Zweden et al. 2011). The positive correlation between spatial and chemical distance for *n*-alkanes was somewhat unexpected, as generally only nest-mate cues are expected to be closely associated with genotype. Nonetheless, a close link between genetic relatedness and *n*-alkane profile also has been found previously (Arnold et al. 1996; Boomsma et al. 2003; and Martin et al. 2012c). This suggests that *n*-alkanes may have a genetic underpinning, but probably not as recognition cues, which may reflect other parts of the genome that are selectively neutral or regulated by different selection regimes, yet associated with genetic similarity. It also suggests that, rather than isolation due to dispersal barriers, divergence may be due to more gradual changes in environment.

Over a period of 3 years, the time of sampling (in 2008 and 2010) explained a small (5 %), but significant, proportion of variation in colony-specific (*Z*)-9-alkene profiles, although the change was non-directional. The findings that 91 % of this variation was explained by colony of origin, and that the between-year relationship was highly significant, again supports the role of (*Z*)-9-alkenes as recognition cues (Châline et al. 2005; Dani et al. 2005; Martin et al. 2011). A similar colony-specific change was not found for the *n*-alkanes, which suggests that environmental factors may affect these compounds more than they affect (*Z*)-9-alkenes, as would be expected if their primary role is protecting ants from the environment (Kather and Martin 2012).

Six of the seven polygynous colonies studied here had a C_23:1_-rich profile, (relative to C_27:1_). This is in agreement with earlier findings from four polygynous populations, containing a total of 37 colonies, chemotyped across Europe; these all had C_23:1_-rich and C_27:1-_poor profiles (Martin et al. 2009). Accordingly, we found a borderline association with social type (mono- vs. poly-gynous). However, we found a stronger association between colony size (i.e., worker population) and (*Z*)-9-alkene profile, and an interaction between social type and colony size, such that more populous colonies had more C_23:1_-rich profiles. Given that polygynous colonies of this species are more populous than monogynous ones (Sundstrom 1995, this study), the profile differences between social types may be, to a large extent, due to colony size rather than to social type, but more data from other populations are needed to test this finding. No association between social type or colony size and cuticular profile was found for the *n*-alkanes.

Although there was an association between colony size and (*Z*)-9-alkene profile, and also among colony size, colony age, and worker size, we found no association between worker size and (*Z*)-9-alkene profile. This most likely is due to the fact that although polygynous colonies are larger, they have relatively smaller workers. We found, however, a small change in the colony-specific profile between 2008 and 2010 for the (*Z*)-9-alkenes. Apart from environmental differences between the two sampling periods, only age and size of the colonies might have changed. As colony age had no effect on either (*Z*)-9-alkene or *n*-alkane profiles, a change in colony size seems likely.

Although all the colonies were on islands with largely similar environmental conditions, there were detectable differences among colonies in their exposure to light, mound temperature, and humidity (Fig. 2). In this study, we found no evidence for an association between CHC and either temperature or humidity. However, the effect sizes were considerable and our sample sizes were low. Hence, no firm conclusions can be drawn at this point on the effects of environmental factors on CHC chemistry. For example, during the summer of 2010, a heat wave caused an abnormally long period of hot and dry conditions across southern Finland. This caused a shift to longer-chain (*Z*)-9-alkenes in all nine *F. exsecta* colonies under study, thereby maintaining the chemical differences among the nine colonies. This change was transitory, as by the autumn of 2010 colony profiles had returned to their pre-summer positions (Martin et al. 2012b). A similar phenomenon previously was seen in the ant *Camponotus aethiops*, with their *n*-alkane profiles changing simultaneously in six colony fragments, despite all being maintained under constant laboratory conditions (van Zweden et al. 2009). Therefore, it appears that environmental conditions can affect CHC profiles, although how it does so is not fully understood. Thus, environment per se can affect *F. exsecta* colony CHC profiles over short time periods, but it may have only a minor role in determining and maintaining diversity of these profiles. This might be expected from an evolutionary perspective of recognition cues, as colonies need to retain identity despite living in an environment that is highly variable over time (Martin et al. 2012b). This is also consistent with the association between genetic and chemical similarity found in this, and an earlier study (van Zweden et al. 2011).

In summary, the conjecture that genetic factors are the most likely source of between-colony variation in CHCs is supported by our study. Both *n*-alkanes and (*Z*)-9-alkenes responded to environmental factors, but often in different ways, indicating that their production is controlled by different genetic pathways. In particular, *n*-alkane profiles showed patterns of variation that corresponded to both population structuring and spatial distance, at scales much greater than those previously found between colonies. This would help explain why there is a large difference between within-colony and between-colony variation in (*Z*)-9-alkenes, but not for *n*-alkanes, and supports the primary role of (*Z*)-9-alkenes as recognition cues and that of *n*-alkanes, and other cuticular lipids, as anti-desiccants.

## Electronic supplementary material

Below is the link to the electronic supplementary material.

ESM 1 (DOC 194 kb)
