# Supplementary material for: Sources of Variation in Cuticular Hydrocarbons in the Ant Formica exsecta
Source: J Chem Ecol. 2013 Nov 24;39(11):1415–23. doi: 10.1007/s10886-013-0366-0 (PMC3851696; doi:10.1007/s10886-013-0366-0)
Supplement: Supplementary file 1 — (DOC 194 kb) [file 10886_2013_366_MOESM1_ESM.doc]

Supplementary material

Table 1 – partitioning of variance among islands and colonies, respectively for each compound.

ANOVAs

Table 2 – comparison between 2008 and 2010, variance components

Table 3 – effects of colony type, age and size on worker size

Table 4 – effects of environmental and colony-specific variables on Z9-alkenes (Factor score 1) a. Full model b. final model

Table 5 – effects of environmental and colony-specific variables on n-alkanes (Factor score 1) a. Full

model b. Final model

Table 6 – Individual components analyses, Z9 alkenes and n-alkanes a. Full model b. Final model

Graph 1 – Effects of humidity and temperature on Z9-alkenes and n-alkanes-

******************************************************************

**Table 1.**

ANOVA results for tests on among-colony and among-island differences in chemical profiles. DFnum is given first and then DFden.

Z9-alkenes C23:1 C25:1 C27:1 C29:1

Factor *DF* *F* *P* *F* *P* *F* *P* *F* *P*

Island 2, 81 1.03 0.36 1.55 0.21 0.89 0.41 1.63 0.20

Colony(isl) 81, 669 105.6 <0.001 125.4 <0.001 266.2 <0.001 85.1 <0.001

n-alkanes C23 C25 C27 C29

Factor *DF* *F* *P* *F* *P* *F* *P* *F* *P*

Island 2, 82 3.87 0.023 21.89 <0.001 0.85 0.43 15.56 <0.001

Colony(isl) 81, 713 20.4 <0.001 13.1 <0.001 18.9 <0.001 11.9 <0.001

**Table 2**

Variance components (%explained)

|  | df | C23 | C25 | C27 | C29 |  | C23:1 | C25:1 | C27:1 | C29:1 |
| --- | --- | --- | --- | --- | --- | --- | --- | --- | --- | --- |
| year | 1 |  |  |  |  |  | 89.8 | 80.7 | 92.8 | 67.4 |
| colony | 31 |  | 21.7 | 28.8 | 26 |  | 0.1 | 0.1 | 0.1 |  |
| Y * C | x | 64.8 | 44.1 | 39.8 | 35.6 |  | 5.7 | 12.7 | 3.8 | 17.2 |
| E |  | 35.2 | 34.2 | 31.4 | 38.4 |  | 4.4 | 6.5 | 3.3 | 15.4 |

Linear regression

| *R*^2^ |  | 0.0003 | 0.1299 | 0.1129 | 0.1651 |  | 0.8841 | 0.7479 | 0.9160 | 0.6239 |
| --- | --- | --- | --- | --- | --- | --- | --- | --- | --- | --- |
| *F*1,3 |  | 0.011 | 4.627 | 3.946 | 6.133 |  | 228.91 | 89.006 | 327.44 | 49.786 |
| *P* |  | 0.918 | 0.039 | 0.056 | 0.019 |  | <0.001 | <0.001 | <0.001 | <0.001 |

**Table 3**

Effects of colony type (mono- or polygynous), age and size on worker size.

| Parameter | estimate | s.e. | *df* | *F* | *P* |
| --- | --- | --- | --- | --- | --- |
| colony size | 0.07 | 0.03 | 68 | 5.48 | 0.022 |
| colony age | 0.52 | 0.14 | 68 | 14.94 | <.001 |
| colony type | -6.32 | 1.84 | 68 | 11.85 | <.001 |
| colony size.colony_age | -0.01 | 0.01 | 65 | 3.14 | 0.081 |
| colony_age.colony type | -0.18 | 0.59 | 65 | 0.09 | 0.760 |
| colony size.colony type | 0.08 | 0.07 | 65 | 1.28 | 0.262 |

**Table 4a**.

Effects of colony-specific variables on the Z9-alkene profile (factor score 1) of colony workers; GLM with sequential dropping of non-significant terms. *indicates terms included in final model. Colony type = monogynous or polygynous. VIF = variation inflation factor. A VIF above five indicates collinearity; as all values are well below this threshold, correlations between explanatory variables are not a concern in this analysis.

| Term | Wald statistic | *d.f.* | *F* statistic | F pr. | VIF |
| --- | --- | --- | --- | --- | --- |
| Island | 3.664 | 2 | 1.83 | 0.169 | - |
| *Colony type | 4.751 | 1 | 4.75 | 0.033 | - |
| Relatedness | 0.468 | 1 | 0.47 | 0.496 | 1.642 |
| colony_age | 0.187 | 1 | 0.19 | 0.667 | 1.344 |
| head_width | 0.082 | 1 | 0.08 | 0.775 | 1.308 |
| *colony_size | 5.969 | 1 | 5.97 | 0.017 | 1.63 |
| shade | 0.376 | 1 | 0.38 | 0.542 | 1.128 |
| Colony type.Island | 2.389 | 1 | 2.39 | 0.127 |  |
| Relatedness.Island | 4.209 | 2 | 2.1 | 0.131 |  |
| colony_age.Island | 5.341 | 2 | 2.67 | 0.077 |  |
| head_width.Island | 3.856 | 2 | 1.93 | 0.154 |  |
| colony_size.Island | 4.4 | 2 | 2.2 | 0.12 |  |
| shade.Island | 0.723 | 2 | 0.36 | 0.698 |  |
| Relatedness.Colony type | 0.519 | 1 | 0.52 | 0.474 |  |
| colony_age.Colony type | 0.219 | 1 | 0.22 | 0.642 |  |
| head_width.Colony type | 0.249 | 1 | 0.25 | 0.62 |  |
| *colony_size.Colony type | 4.768 | 1 | 4.77 | 0.033 |  |
| Colony type.shade | 1.2862 | 1 | 1.29 | 0.261 |  |
| Relatedness.colony_age | 0.067 | 1 | 0.07 | 0.797 |  |
| Relatedness.head_width | 0.437 | 1 | 0.44 | 0.511 |  |
| colony_size.Relatedness | 0.9939 | 1 | 0.99 | 0.322 |  |
| Relatedness.shade | 2.391 | 1 | 2.39 | 0.127 |  |
| colony_age.head_width | 0.207 | 1 | 0.21 | 0.651 |  |
| colony_age.colony_size | 1.182 | 1 | 1.18 | 0.281 |  |
| colony_age.shade | 1.63 | 1 | 1.63 | 0.207 |  |
| head_width.colony_size | 0.214 | 1 | 0.21 | 0.645 |  |
| head_width.shade | 0.163 | 1 | 0.16 | 0.688 |  |
| colony_size.shade | 0.586 | 1 | 0.59 | 0.447 |  |

**Table 4b.**

Effects of colony specific variables on Z9-alkene factor scores; final model
Regression *F* _3, 71_ = 10.44, *P*‹ 0.001; accounting for 27.7% of the variation.

| Parameter | estimate | s.e. | t(71) | t pr. |
| --- | --- | --- | --- | --- |
| Constant | 0.128 | 0.103 | 1.24 | 0.219 |
| colony size | -0.532 | 0.142 | -3.74 | <.001 |
| type | -1.095 | 0.404 | -2.71 | 0.008 |
| colony size.type | 0.475 | 0.228 | 2.09 | 0.041 |

**Table 5a.**

Effects of colony-specific variables on the *n*-alkane factor score 1 of colony workers. GLM with sequential dropping of non-significant terms, * indicates terms included in final model. Colony type = monogynous or polygynous.

| Factor | Wald statistic | *d.f.* | *F* statistic | F pr. | VIF |
| --- | --- | --- | --- | --- | --- |
| *Island | 21.14 | 2 | 10.57 | <0.001 | - |
| Colony type | 0.027 | 1 | 0.03 | 0.871 | - |
| Relatedness | 0.181 | 1 | 0.18 | 0.672 | 1.642 |
| colony_age | 0.001 | 1 | 0 | 0.98 | 1.344 |
| head_width | 1.306 | 1 | 1.31 | 0.258 | 1.308 |
| colony_size | 1.236 | 1 | 1.24 | 0.271 | 1.63 |
| shade | 0.695 | 1 | 0.7 | 0.408 | 1.128 |
| Relatedness.Island | 1.3655 | 2 | 0.68 | 0.509 |  |
| Relatedness.Colony type | 0.338 | 1 | 0.34 | 0.563 |  |
| Relatedness.colony_age | 0.548 | 1 | 0.55 | 0.462 |  |
| Relatedness.head_width | 0.407 | 1 | 0.41 | 0.526 |  |
| Relatedness.colony_size | 0.09 | 1 | 0.09 | 0.765 |  |
| Relatedness.shade | 0.006 | 1 | 0.01 | 0.94 |  |
| Island.Colony type | 0.6381 | 1 | 0.64 | 0.427 |  |
| colony_age.Island | 5.649 | 2 | 2.82 | 0.067 |  |
| head_width.Island | 0.4595 | 2 | 0.23 | 0.795 |  |
| colony_size.Island | 3.1031 | 2 | 1.55 | 0.22 |  |
| shade.Island | 3.234 | 2 | 1.62 | 0.207 |  |
| colony_age.Colony type | 0.005 | 1 | 0.01 | 0.942 |  |
| colony_size.Colony type | 0.058 | 1 | 0.06 | 0.811 |  |
| head_width.Colony type | 0.885 | 1 | 0.89 | 0.35 |  |
| shade.Colony type | 0.001 | 1 | 0 | 0.977 |  |
| colony_age.colony_size | 2.848 | 1 | 2.85 | 0.097 |  |
| colony_age.shade | 0.795 | 1 | 0.79 | 0.376 |  |
| colony_age.head_width | 0.139 | 1 | 0.14 | 0.71 |  |
| colony_size.shade | 1.458 | 1 | 1.46 | 0.232 |  |
| colony_size.head_width | 0.733 | 1 | 0.73 | 0.395 |  |
| *shade.head_width | 5.22 | 1 | 5.22 | 0.025 |  |

**Table 5b**.

Effects of colony specific variables on *n*-alkene factor score 1; final model
Regression *F* _3, 68_ = 7.86, *P* ‹ 0.001; accounting for 22.5% of the variation.
Factor Island levels calculated in reference to island Furuskär

| Parameter | estimate | s.e. | t(68) | t pr. |
| --- | --- | --- | --- | --- |
| Constant | 1.378 | 0.401 | 3.44 | <.001 |
| Relatedness | -1.663 | 0.552 | -3.01 | 0.004 |
| Island: Joskar | -0.777 | 0.215 | -3.62 | <.001 |
| Island: Rovholmen | -0.393 | 0.254 | -1.55 | 0.126 |
|  |  |  |  |  |

**Table 6**: Individual components analysis summary

| a) Z9 alkenes: |  | C23:1 |  | C25:1 |  | C27:1 |  | C29:1 |  |
| --- | --- | --- | --- | --- | --- | --- | --- | --- | --- |
| individual components summary | *df* | *F* | *P* | *F* | *P* | *F* | *P* | *F* | p |
| std_colony_size | 1 | 7.71 | 0.007 | 3.87 | 0.054 | 5.55 | 0.022 | 2.88 | 0.095 |
| Island | 2 | 2.03 | 0.14 | 4.68 | 0.013 | 0.73 | 0.486 | 1.94 | 0.152 |
| Relatedness | 1 | 0.25 | 0.619 | 0.32 | 0.571 | 1.59 | 0.211 | 0.09 | 0.768 |
| colony_age | 1 | 0.02 | 0.898 | 0.05 | 0.818 | 0.79 | 0.379 | 0.22 | 0.64 |
| std_shade | 1 | 0.11 | 0.743 | 0.85 | 0.36 | 0.34 | 0.559 | 0.03 | 0.869 |
| std_HW | 1 | 0.05 | 0.822 | 1.35 | 0.25 | 0.04 | 0.841 | 0.39 | 0.537 |
| Mono_v_poly | 1 | 3.57 | 0.063 | 4.12 | 0.047 | 7.37 | 0.009 | 0.56 | 0.458 |
| Residual d.f. 62 |  | | | | | | | | |
| All interactions p›0.08 |  | | | | | | | | |

b) *n*-alkanes:

|  |  | C23 |  | C25 |  | C27 |  | C29 |  |
| --- | --- | --- | --- | --- | --- | --- | --- | --- | --- |
| Term | *df* | *F* | *P* | *F* | *P* | *F* | *P* | *F* | p |
| Island | 2 | 5.41 | 0.007 | 12.99 | <0.001 | 0.93 | 0.402 | 11.79 | <0.001 |
| std_colony_size | 1 | 0.75 | 0.391 | 0.52 | 0.474 | 1.81 | 0.183 | 1.02 | 0.317 |
| std_shade | 1 | 0.1 | 0.753 | 1.06 | 0.307 | 0.01 | 0.943 | 1.99 | 0.164 |
| std_HW | 1 | 1.47 | 0.23 | 0.21 | 0.65 | 0 | 0.982 | 0.13 | 0.718 |
| Relatedness | 1 | 3.05 | 0.086 | 3.89 | 0.053 | 4.21 | 0.044 | 17.85 | <0.001 |
| colony_age | 1 | 0.51 | 0.478 | 2.94 | 0.092 | 1.81 | 0.184 | 4.28 | 0.043 |
| Mono_v_poly | 1 | 1.23 | 0.272 | 0.78 | 0.38 | 0.21 | 0.645 | 9.25 | 0.003 |

Residual df 62, all interactions p›0.09

Supplementary figure 1.
Relationships between Z9 –alkene and *n*-alkane factor scores, temperature (a-b) and humidity (c-d).
